# Quality improvement initiative to reduce late-onset sepsis in very low birth weight preterm infants: a multicenter study from the Brazilian network on neonatal research

**DOI:** 10.1016/j.jped.2025.101459

**Published:** 2025-10-15

**Authors:** Ligia Maria Suppo de Souza Rugolo, Maria Regina Bentlin, Fernanda Pegoraro de Godoi Melo, Maria Eduarda Gurgel, Gabriella Miranda Martins, Suely Dornellas do Nascimento, Edna Maria de Albuquerque Diniz, Renata Yoshida, Jamil Pedro de Siqueira Caldas, Silvia Cwajg, Marcia Pereira Alves de Souza, Clarissa Gutierrez Carvalho, Daniela Marques de Lima Mota Ferreira

**Affiliations:** a Universidade Estadual Paulista Júlio de Mesquita Filho (UNESP), Faculdade de Medicina de Botucatu, Departamento de Pediatria, Disciplina de Neonatologia, São Paulo, SP, Brazil; b Universidade Estadual de Londrina (UEL), Departamento de Pediatria, Disciplina de Neonatologia, Londrina, PR, Brazil; c Instituto de Medicina Integral Professor Fernando Figueira (IMIP), Serviço de Neonatologia, Recife, PE, Brazil; d Universidade Federal do Maranhão (UFMA), Departamento de Pediatria, Disciplina de Neonatologia, São Luis, MA, Brazil; e Universidade Federal de São Paulo (UNIFESP), Escola Paulista de Medicina, Departamento de Pediatria, Disciplina de Pediatria Neonatal, São Paulo, SP, Brazil; f Hospital Universitário da Faculdade de Medicina da Universidade de São Paulo (USP), São Paulo, SP, Brazil; g Faculdade de Medicina da Universidade de São Paulo (FMUSP), Departamento de Pediatria, Divisão de Neonatologia, São Paulo, SP, Brazil; h Hospital das Clínicas da Faculdade de Medicina da Universidade de São Paulo (HCFMUSP), São Paulo, SP, Brazil; i Universidade Estadual de Campinas (UNICAMP), Departamento de Pediatria, Divisão de Neonatologia, Campinas, SP, Brazil; j Instituto Fernandes Figueira/ Fiocruz, Divisão de Neonatologia, Rio de Janeiro, RJ, Brazil; k Universidade Estadual do Rio de Janeiro (UERJ), Divisão de Neonatologia, Rio de Janeiro, RJ, Brazil; l Universidade Federal do Rio Grande do Sul (UFRGS), Departamento de Pediatria, Divisão de Neonatologia, Porto Alegre, RS, Brasil; m Universidade Federal de Uberlândia (UFU), Departamento de Pediatria, Divisão de Neonatologia, Uberlândia, MG, Brasil

**Keywords:** Sepsis, Quality improvement, Premature infants, Infant, Very low birth weight

## Abstract

**Objective:**

To evaluate the impact of a quality improvement project (QI) on reducing proven late-onset sepsis (LOS) in centers of the Brazilian Network Neonatal Research (BNNR).

**Method:**

An interventional study conducted in 12 BNNR centers from 2021 to 2023. Included preterm infants (PT) born at 22–36 weeks' gestational age, weighing 400–1499 grams, without malformations, and admitted to the NICU for > 72 h. QI tools were used and four process indicators were defined: central catheter complication (≤ 20 %); antibiotic discontinuation ≤48 h in non-infected infants (≥ 80 %); breast milk expression within the first 48 h and enteral feeding within the first 24 h of life (≥ 80 %); full enteral feeding without parenteral nutrition by day 11 (≥ 70 %). The outcome was the proportional reduction of LOS according to each center’s baseline (2020). Indicators were analyzed descriptively across three periods.

**Results:**

A total of 1993 PT < 1500 grams were included. Half of the centers achieved the target for umbilical catheter complications, and 92 % for percutaneous catheters. Antibiotics were discontinued within 48 h in 67 % of non-infected infants. Early breast milk expression and enteral feeding were achieved in 44 % and 75 % of cases, respectively. 58 % achieved full enteral nutrition without parenteral support by day 11. LOS incidence declined in 67 % of centers, and half met their targets, with an overall 18.5 % reduction.

**Conclusions:**

The project reduced LOS in most centers, although some clinical practices still need improvement. It demonstrates a reproducible, low-cost strategy with the potential to guide other neonatal units facing high sepsis incidence.

## Introduction

Late-onset sepsis (LOS), especially in very low birth weight (VLBW) preterm infants, remains a major challenge in neonatal intensive care units (NICUs) [1,2]. A Vermont Oxford Network study involving 118,650 infants born at 22–29 weeks’ gestation and weighing between 401 and 1500 g, reported a proven LOS incidence of 8.9 %, reaching 21.9 % in 24–25 weeks and 32 % under 24 weeks. Septic preterm infants (PT) had lower survival rates (78,2 %) compared to non-septic (94,9 %), and higher need for home oxygen, tracheostomy, and gastrostomy, conditions that impact quality of life [3].

In Brazil, the situation is more challenging. A study of the Brazilian Network on Neonatal Research (BNNR), involving 13,439 PT born between 22 and 36 weeks of gestation and weighing between 400 and 1499 g, from 2010 to 2020, reported a proven LOS incidence of 24.6 %, ranging between centers from 13 % to 49 %. The mortality rate was 23.5 %, with sepsis being the terminal cause of death in 81 % of these infants [4]. These findings are consistent with data published by the BNNR in 2014, indicating no improvement over the years [5].

Incorrect hand hygiene, improper and excessive manipulation of central lines, delayed initiation of enteral feeding, prolonged use of parenteral nutrition, mechanical ventilation, and indiscriminate antibiotic administration have been identified as factors associated with LOS [4, 5, 6, 7, 8, 9]. These practices can be optimized through quality improvement (QI) initiatives aimed at enhancing the efficiency of the processes.

Quality improvement initiatives enable protocol standardization, team training, continuous monitoring, and outcome evaluation, fostering a safer environment centered on preventive practices and enhanced teamwork [2,10]. In Brazil, initiatives to reduce LOS have been restricted to single-center efforts, with no national guideline available. Since the incidence of LOS remains high in BNNR NICUs, with sepsis being the leading cause of preventable death in preterm infants and closely linked to care practices, the BNNR implemented for the first time a quality improvement project aiming to reduce LOS by enhancing clinical care practices, thereby contributing to better neonatal outcomes through a low-cost, technology-free intervention reproducible in all neonatal units.

## Method

This was a multicenter interventional study conducted in 12 NICUs participating in the BNNR, between May 2021 and December 2023. In 2020, the BNNR reported one of the highest rates of culture-proven LOS, approaching 30 %. Then, the network’s coordinating committee prioritized the reduction of LOS and designated one of its centers to lead a QI initiative, entitled **Down Late-Onset Sepsis (DownLOS)**.

### DownLOS project

Of the 20 centers invited, 12 agreed to participate in the project. The study was approved by the ethics committee of the coordinating center, and a formal agreement was obtained from all participating institutions (CAAE: 79,997,224.3.0000.5411).

The project was structured into five phases, implemented through bimonthly online meetings.

• **Phase 1 (January 2021):** Contextualization of LOS within the BNNR and presentation of the methodological framework, based on QI tools including: PDCA cycle (Plan: problem identification and action plan; Do: implementation; Check: monitoring; Act: standardization), Ishikawa diagram (to identify potential causes of sepsis), Pareto chart (to prioritize interventions), and 5W2H tool (What, Why, Who, Where, When, How, How much) for each standardized action. Considering the heterogeneity of centers regarding infrastructure, human resources, and care practices, two questionnaires addressing these aspects were distributed to the representatives of each center. Additionally, a target for proportional reduction in proven LOS was agreed upon, based on each center's 2020 baseline rate.
• **Phase 2 (February 2021):** On-site situational assessment was conducted at each center, including team engagement, identification of the main contributing factors to LOS, and completion of the questionnaires.
• **Phase 3 (March 2021):** Selection of process and outcome indicators, as well as prioritization of actions. Four process indicators were defined based on previous RBPN studies [5,6], and the centers’ consensus on causes and actions from Ishikawa and Pareto analyses.

1. Incidence of central line–related complications.
2. No use or discontinuation of empirical antibiotics (within 48 h) in preterm infants at risk for early-onset sepsis but without confirmed infection.
3. Use of breast milk and early initiation of enteral nutrition.
4. Full enteral feeding without parenteral nutrition in preterm infants ≥ 1000 g by 11 days of life.

The outcome indicator was the proportional reduction in the rate of proven LOS.

• **Phase 4 (April 2021):** Development of action plans at each center, guided by the 5W2H.
• **Phase 5 (May 2021 to December 2023):** Implementation of the project. Data were collected using standardized spreadsheets and submitted to the coordinating center, which compiled and anonymized the information. Process indicators were analyzed and shared with all participating centers, fostering collective discussions and adjustments through new PDCA cycles, as needed. The incidence of proven LOS was monitored annually.

Project implementation was stratified into three periods:

• P1 – Initial Implementation Phase (May to December 2021): Launch of local interventions based on the action plans.
• P2 – First PDCA Cycle (January to December 2022): Evaluation and refinement of strategies based on process indicators.
• P3 – Second PDCA Cycle (January to December 2023): Further adjustments and consolidation of effective practices.

### Inclusion and exclusion criteria for newborns

All preterm infants of 22–36 weeks’ gestation, weighing 400–1499 g, inborn or outborn, hospitalized in the NICU for >72 h, were included. Newborns with major congenital malformations were excluded.

### Indicators and targets

**1. Central Line Complications**

**  ***○ Indicator:* Number of central venous catheters (umbilical or peripherally inserted central catheters [PICC]) removed due to complications ÷ Total number of catheters inserted.

**  ***○ Target:* ≤ 20 %.

**2. No use or discontinuation of antibiotics ≤ 48 h in infants at risk of early-onset sepsis after infection was ruled out**

**  ***○ Indicator:* Number of newborns who did not receive antibiotics or had antibiotics discontinued within 48 h in the context of infectious risk ÷ Total number of newborns in infectious risk situations without confirmed infection.

**  ***○ Target:* ≥ 80 %

**3. Breast milk expression ≤48hours after delivery and beginning enteral nutrition ≤ 24hours of life**

**  **• Indicator (Breast milk expression): Number of mothers who expressed colostrum within the first 48 h after delivery ÷ Total number of mothers eligible to breastfeed.

**  **• Target: ≥ 80 %

**  **• Indicator (Early enteral nutrition): Number of newborns who received enteral feeding within the first 24 h of life ÷ Total number of VLBW newborns.

**  **• Tar*get:* ≥ 80 %

 **4. Full enteral feeding without parenteral nutrition in preterm infants ≥ 1000 *g* by 11 days of life**.

**  ***• Indicator:* Number of newborns with birth weight ≥ 1000 g who achieved full enteral nutrition without parenteral support by the 11th day of life ÷ Number of newborns with birth weight ≥ 1000 g who survived beyond 10 days of life.

**  ***• Target:* ≥ 70 %

### Outcome indicator

The primary outcome indicator was the annual incidence of proven LOS.

**  ***• Indicator:* Number of newborns with proven LOS, diagnosed by blood culture ÷ Total number of VLBW newborns admitted to the NICU for >72 h.

**  ***• Target:* Proportional reduction in incidence, relative to the 2020 baseline. Centers with LOS:

**  **○ > 40 % → Reduce to 30–40 %.

**  **○ 30–40 % → Reduce to 20–30 %.

**  **○ 20–30 % → Reduce to 10–20 %.

**  **○ 10–20 % → Reduce to < 10 %.

Sepsis caused by coagulase-negative staphylococci was defined according to BNNR criteria: one or more positive blood cultures, associated with clinical and laboratory signs of infection, and antibiotic therapy for ≥ 5 days. Micrococcus sp., *Corynebacterium* sp., and *Propionibacterium* sp. were considered contaminants.

### Statistical analysis

Data were expressed by means and standard deviation, number and proportions of events, and summarized in frequency and association tables. To evaluate the achievement of process indicators targets, comparisons were made between the first and third periods. For the outcome indicator- proportional LOS reduction- targets were predefined by consensus according to each center’s incidence in 2020, and the baseline data from 2020 were compared with those from the third period.

## Results

Between May 2021 and December 2023, a total of 2247 very low birth weight preterm infants were admitted to the 12 BNNR centers participating in the DownLOS project. Of these, 254 infants were excluded for not meeting the inclusion criteria. The final study sample comprised 1993 infants (flowchart below).

**Figure:**
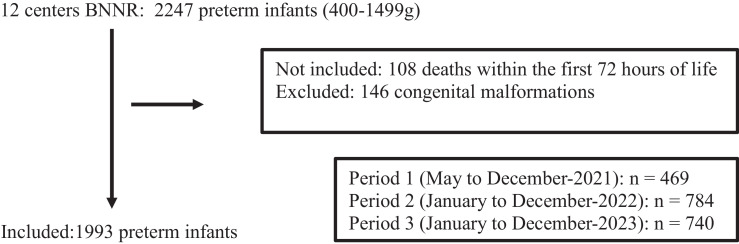
Flowchart of the DownLOS project.

### Questionnaires on practices and infrastructure

All 12 centers had established protocols or bundles for central line insertion. Both the medical team responsible for placing umbilical lines and the nursing team inserting PICCs had received technical training. All centers had reviewed their protocols for early-onset infection and infectious risk, and all had standardized enteral nutrition protocols. Regarding the investigation of LOS, 67 % of the centers reported collecting two blood cultures, 92 % initiated empirical antibiotic therapy with oxacillin and amikacin, discontinued treatment after 5–7 days for clinical sepsis and 7–10 days for culture-proven sepsis. Table 1 summarizes the main data on infrastructure and human resources across the centers.

**Table 1:** Main infrastructure and human resources characteristics of the 12 centers participating in the DownLOS project.

Centers	N^o^ PT	N° NICU beds	N° bed/nursing technicians	N° bed/ supervised by nurse		% nurses specialized Neonatalogy	NICU occupancy rate (%)
1#	44	4	2	4	<30		63
2⁎⁎	203	17	3	10–15	30–50		90
3#	172	20	2–3	4–5	30–50		100
4*	381	35	2	30	<30		95
5*	194	20	2	20	30–50		95
6#	194	40	3	10	>50		90
7#	263	20	2–3	10	>50		95
8⁎⁎	60	14	3–4	8	>50		91
9⁎⁎	50	12	3	7	>50		90
10⁎⁎	180	10	2–3	5–8	>50		>100
11⁎⁎	68	16	3	10	>50		68
12⁎⁎	184	15	3	15	30–50		85

⁎⁎ Centers that achieved the target for LOS reduction.

⁎ Centers that improved but did not reach the target.

# Centers that did not improve. N°, number; NICU, neonatal intensive care unit; LOS, late-onset sepsis.

### Process and outcome indicators

**Fig. 1:**
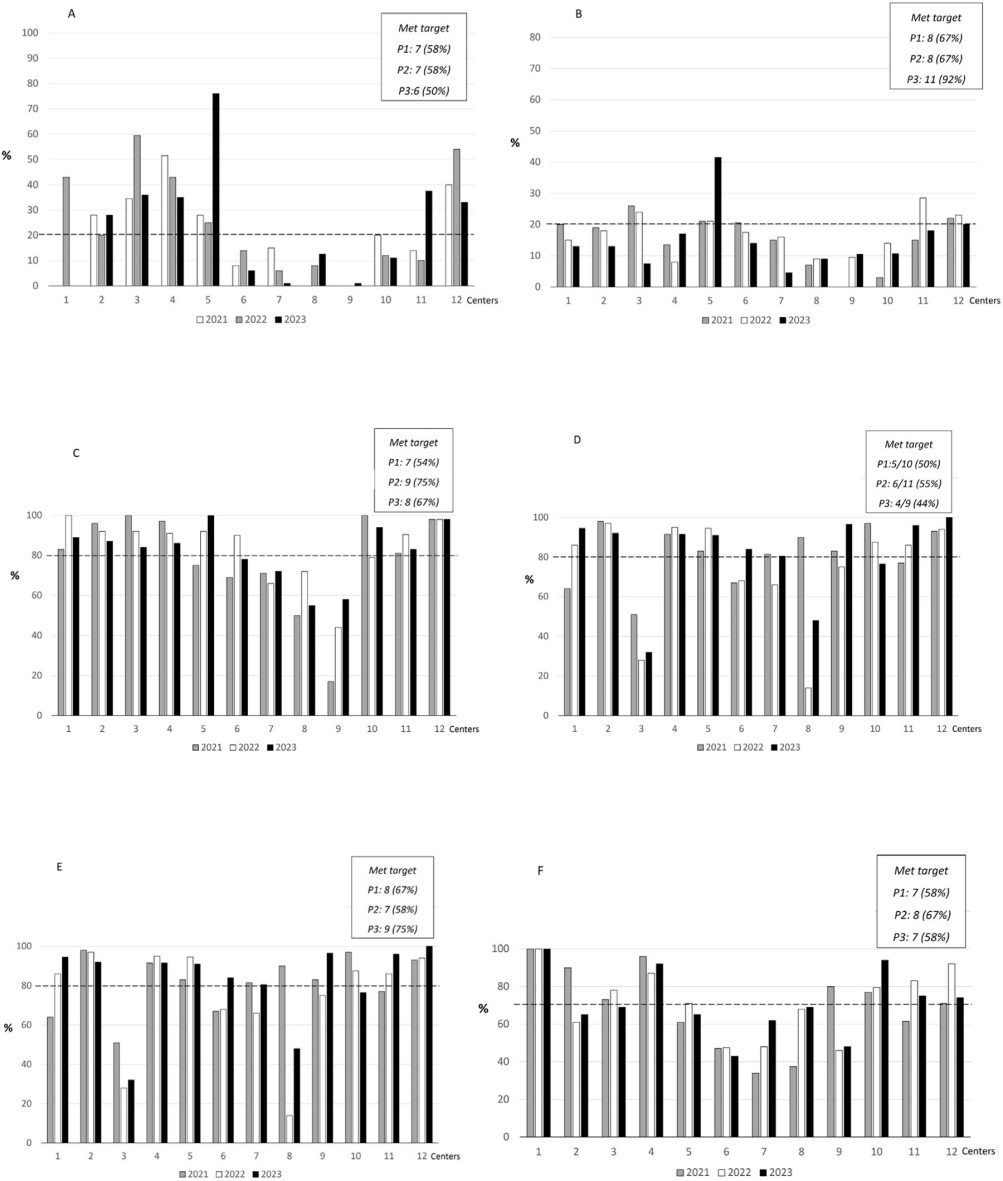
Process indicators in the 12 BNNR centers across the 3 study periods (P). **(A)** Umbilical catheter complications. **(B)** Peripherally inserted central catheter (PICC) complications. **(C)** No use or discontinuation of antibiotics ≤48 h in infants at risk of early-onset sepsis after infection was ruled out. **(D)** Breast milk expression ≤ 48 h of life. **(E)** Beginning enteral nutrition ≤ 24 h of life. **(F)** Full enteral feeding without parenteral nutrition in preterm infants ≥ 1000 g by 11 days of life. BNNR, Brazilian Network on Neonatal Research; P, period; Dashed line, process indicator target.

Figure 1 presents the evolution of process indicators. The indicators with the least satisfactory performance included unscheduled removal of umbilical catheters and breast milk expression within the first 48 h after delivery. Although several centers showed improvement in the early initiation of enteral nutrition, only a few managed to reduce the duration of parenteral nutrition to 10 days or less.

Regarding the outcome indicator, most centers showed a reduction in LOS incidence, with half achieving the target (Table 2, Figure 2). Across the 12 centers, incidence declined from 27 % (95 % CI: 21–33) in 2020 to 22 % (95 % CI: 15–30) in 2023, an 18.5 % relative reduction.

**Table 2:** Incidence of proven late-onset sepsis (LOS) by center in the baseline year 2020; proposed target, incidence in 2023 and percentage change from 2020 to 2023.

Centers	LOS 2020 (%)	Target (%)	LOS 2023 (%)	% Change
1 #	11.5	<10	16.7	+45.2
2 ⁎⁎	30.0	20–30	15.8	−47.30
3 #	35.7	20–30	44.4	+24.4
4 *	16.9	<10	14.7	−13.0
5 *	25.6	10–20	24.7	−3.5
6 #	24.3	10–20	28.9	+18.9
7 #	33.3	20–30	35.6	+6.9
8 ⁎⁎	20.0	10–20	19.0	−5.0
9 ⁎⁎	30.4	20–30	10.7	−64.8
10 ⁎⁎	47.0	30–40	36.1	−23.2
11 ⁎⁎	29.3	10–20	12.0	−59.0
12 ⁎⁎	18.9	<10	6.6	−65.1

⁎⁎ centers that achieved the target for LOS reduction.

⁎ centers that improved but did not reach the target.

# centers that did not improve.

**Fig. 2:**
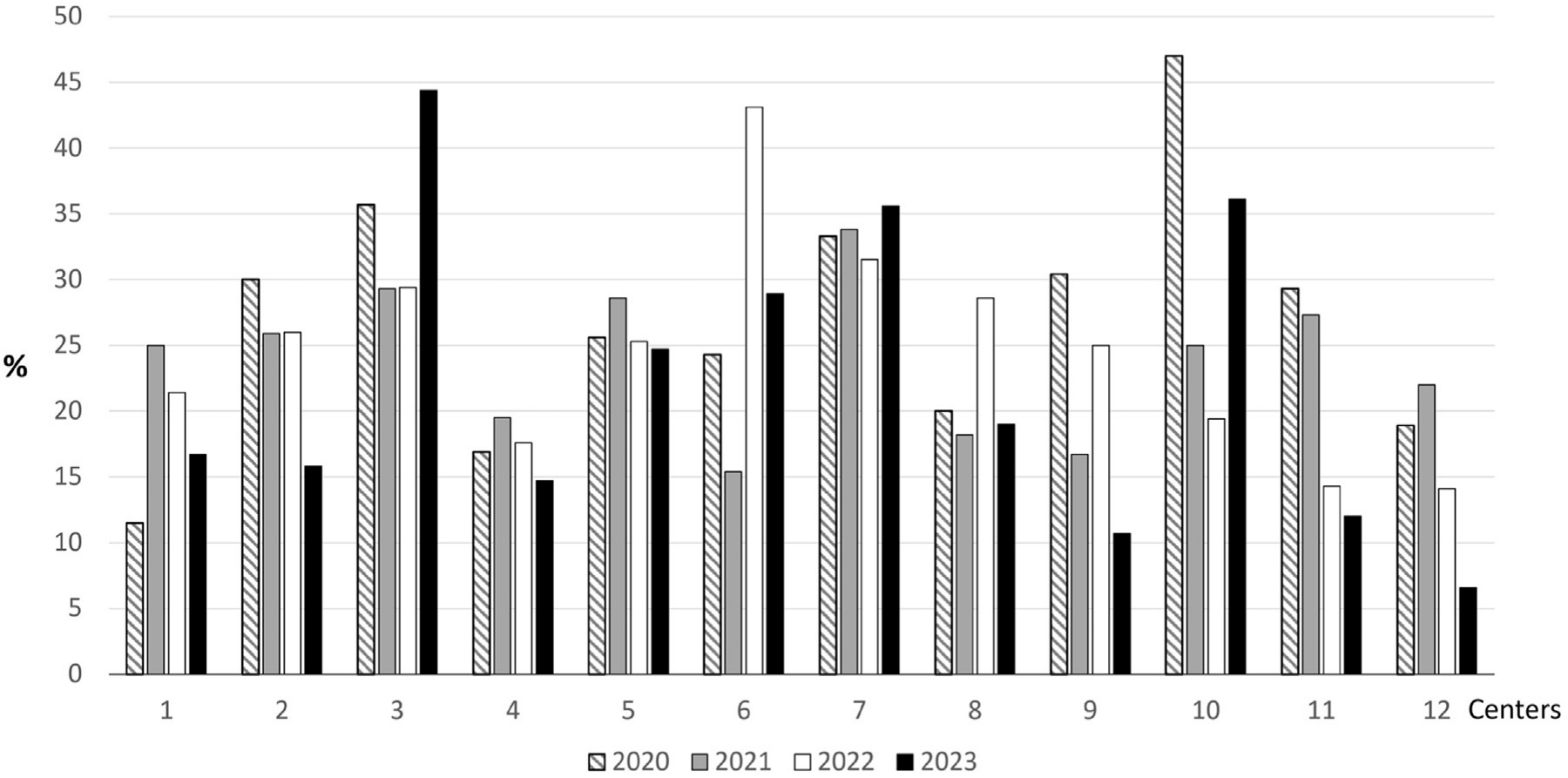
Incidence of proven late-onset sepsis (LOS) by center in the baseline year (2020) and across the three study periods (2021, 2022, 2023).

## Discussion

In high-income countries, multicenter studies have reported an incidence of LOS ranging from 5.3 % to 11.9 % among VLBW newborns [3]. In contrast, LOS incidence has remained largely unchanged in middle- and low-income countries [11].

BNNR data highlight the persistent challenge, with no improvement over the past decades. Proven LOS incidence was 24 % in 2006–2008 and remained 24.6 % in 2010–2020, with risk factors largely linked to healthcare practices [4,6]. The high LOS incidence in the BNNR motivated the DownLOS project, as QI initiatives are cost-effective strategies for improving clinical care. A distinctive feature of this BNNR initiative was its emphasis not only on regular local team meetings but also on inter-institutional presentations and discussions coordinated by the project leadership, promoting the exchange of experiences and encouraging the teams to pursue improved clinical outcomes.

### Questionnaires on practices and infrastructure

Questionnaire responses indicated that all centers had revised their clinical protocols, a positive outcome. However, one-third collected only a single blood culture when investigating LOS. The collection of two blood cultures is essential to distinguish between contamination/colonization and true infection, especially with coagulase-negative staphylococci [12,13]. This practice requires improvement across BNNR centers.

The current infrastructure of the NICUs is a matter of concern. Seventy-five percent of the units reported occupancy rates of ≥ 90 %, often coupled with shortages in human resources (one nursing technician being responsible for at least three beds). In 60 % of centers, nursing supervisors oversee ≥ 10 beds. Furthermore, more than half of the centers rely on informal, peer-based daily training of nursing professionals. These findings contrast with guidelines from the Brazilian Ministry of Health [14,15], which recommend one nursing technician for every two NICU beds and one nurse supervisor for every five to ten beds. Factors such as overcrowded units, staff shortages, excessive workloads, and insufficient training or professional qualifications contribute to failures in care processes.

In high-income countries, neonatal intensive care is typically delivered by nurses specialized in neonatology, a factor that has been associated with reductions in neonatal morbidity [9]. Although this standard is not widely implemented in low- and middle-income countries, Brazil has reported successful initiatives, such as the *Portal de Boas Práticas-Fiocruz*, that offers online courses and educational content aimed at improving the training of neonatal care professionals and fostering the adoption of evidence-based practices [16].

### Process and outcome indicators

Vascular catheters are among the primary risk factors associated with LOS. Unscheduled removal is particularly concerning, not only due to its potential association with infection but also because it necessitates repeated vascular access attempts [17, 18, 19]. Umbilical catheters had the highest rate of unscheduled removals since the beginning of the DownLOS project, and only 50 % of participating NICUs were able to meet the goal. Despite all umbilical catheters being inserted by trained staff, malposition was frequent, and the complication rates remained high, likely due to manipulation or non-standardized fixation techniques, highlighting the need for specific strategies to improve this indicator. A review study on umbilical catheters reported that only 25 % were correctly positioned [18]. Malposition is associated with infections, venous thrombosis, and hepatic and cardiac injury [17,18]. Current best practices recommend planned and timely removal of central lines to prevent catheter-associated bloodstream infections [19].

QI initiatives targeting central vascular catheters have demonstrated promising results.

In a high-income country, one project reduced catheter-associated infections from 8.4 to 1.8 per 1000 catheter-days and catheter-related complications from 47 % to 10 % [20]. Similarly, a low-income country study showed benefits from implementing hand hygiene protocols and catheter care bundles. These interventions reduced catheter-associated infections by 89 %, bloodstream infections from 7.3 to 2.3 per 1000 patient-days, and also reduced mortality [21]. The Society for Healthcare Epidemiology of America recommends the routine use of checklists and care bundles for the insertion and maintenance of central lines [2].

A notable positive outcome of this study was that two-thirds of the centers reduced antibiotic use in the first 72 h for infectious risk, and in over 80 % of newborns, antibiotics were stopped within 48 h when infections were unconfirmed. Suspicion of sepsis is highly prevalent in preterm infants, often prompting treatment based on clinical and laboratory findings that may be attributable to other morbidities, leading to unnecessary antibiotic use [13]. In the BNNR between 2010 and 2020, 54 % of infants received antibiotics in the first three days, while proven early-onset sepsis occurred in only 1.5 %, underscoring the need to reassess this practice [4].

Inappropriate antibiotic use during the first three days of life is associated with adverse outcomes, including pain, stress, dysbiosis, antimicrobial resistance, prolonged hospitalization, and increased risk of LOS and necrotizing enterocolitis [4,5,22]. Guidelines from the American Academy of Pediatrics [23], the Ibero-American Consensus on Sepsis [13], and the Brazilian Society of Pediatrics [24] recommend re-evaluating antibiotic therapy within 36 to 48 h after initiation due to risk factors or clinical presentation. Antibiotics should be discontinued if clinical evolution is favorable and blood cultures remain negative. A QI initiative by the Vermont Oxford Network demonstrated a 24 % reduction in antibiotic use under these situations without an increase in sepsis rates [8].

Regarding nutritional indicators, fewer than half of centers met early breast milk expression targets, with no improvement, while early enteral nutrition increased in 75 % of centers by the third period. However, only slightly more than half of the centers met the goal of reducing the duration of parenteral nutrition, suggesting a slow progression in feeding that prolongs the use of central catheters. Nutritional indicators improvements may result from supporting mothers in milk expression and raising staff awareness of the importance of early introduction and progression of feeding [25].

A QI initiative showed a 30 % reduction in the time to reach full enteral feeding, a 44 % decrease in central catheter days, and a 42 % reduction in parenteral nutrition days in preterm infants. The key drivers of these improvements included the updating of nutritional protocols, obtaining early human milk, and initiating enteral feeding within 12 h of life [26].

In Brazil, breastfeeding and the use of a mother’s own milk are strongly encouraged. The Brazilian Society of Pediatrics’ Neonatal Resuscitation Program emphasizes the “golden hour,” prioritizing skin-to-skin contact and breastfeeding within the first hour of life, as well as the kangaroo care method [27]. These best practices are expected to increase the use of human milk. Early provision of breast milk has been shown to reduce the duration of central line use and parenteral nutrition, while also decreasing the risk of LOS and length of hospitalization [28].

The outcome indicator, proportional reduction in LOS, demonstrated that eight centers (67 %) showed improvement, with six centers meeting the predefined target. This is a highly encouraging result, particularly considering that five of the six centers achieving the goal had a reduction in LOS greater than 20 %.

The DownLOS project has certain limitations. It was a descriptive study without statistical adjustments for center-level differences or patient characteristics, which reduces the ability to isolate the intervention's effect. The project involved 12 centers within the BNNR with variability in infrastructure, number of included patients, and self-reported practices. The outcome definition restricted to culture-proven sepsis may underestimate the true burden of infection. Additionally, variability in action plans across centers may have had some influence on the results. Process indicators may have had variable impacts across centers due to differences in baseline LOS rates. Moreover, the project duration may have been insufficient for some centers to fully implement changes and reach the intended outcomes, and the three-year period may be too short to assess the sustainability of the changes.

Nevertheless, the Project presents several strengths. It represents the first multicenter QI initiative aimed at reducing neonatal LOS in Brazil, with a large patient cohort, and the participation of multiple centers across different regions of a middle-low-income country supports the study’s external validity. The approach involved low-cost interventions and systematic process review. Most notably, the high level of team engagement was a key factor, suggesting the sustainability of this quality initiative, the potential to transform LOS management and to serve as a model for other low- and middle-income countries.

Implementation of the DownLOS project in BNNR centers reduced LOS incidence in two-thirds of units, with half meeting their targets. The results highlight areas for further improvement — such as reducing unscheduled umbilical catheter removals, promoting earlier breast milk expression, and accelerating enteral feeding—supporting the continuation of the project. QI initiatives like DownLOS are essential for safer, evidence-based neonatal care and effective LOS reduction.

## Funding source

Nothing to declare.

## Conflicts of interest

The authors declare no conflicts of interest.
